# Sensorimotor integration is affected by acute whole-body vibration: a coherence study

**DOI:** 10.3389/fphys.2023.1266085

**Published:** 2023-09-12

**Authors:** E. Lecce, S. Nuccio, A. Del Vecchio, A. Conti, A. Nicolò, M. Sacchetti, F. Felici, I. Bazzucchi

**Affiliations:** ^1^ Department of Movement, Human, and Health Sciences, Laboratory of Exercise Physiology, University of Rome “Foro Italico”, Rome, Italy; ^2^ Department Artificial Intelligence in Biomedical Engineering, Faculty of Engineering, Zentralinstitut für Medizintechnik (ZIMT), Friedrich-Alexander University Erlangen-Nürnberg, Erlangen, Germany

**Keywords:** coherence, HDsEMG, motor unit, sensorimotor integration, whole-body vibration

## Abstract

**Introduction:** Several whole-body vibration (WBV) effects on performance have been related to potential changes in the neural drive, motor unit firing rate, and sensorimotor integration. In the present paper, motor unit coherence analysis was performed to detect the source of neural modulation based on the frequency domain.

**Methods:** Thirteen men [25 ± 2.1 years; Body Mass Index (BMI) = 23.9 ± 1.3 kg m2; maximal voluntary force (MVF): 324.36 ± 41.26 N] performed sustained contractions of the Tibialis Anterior (TA) at 10%MVF before and after acute WBV. The vibrating stimulus was applied barefoot through a platform to target the TA. High-Density surface Electromyography (HDsEMG) was used to record the myoelectrical activity of TA to evaluate coherence from motor unit cumulative spike-trains (CSTs).

**Results:** Mean coherence showed a significant decrease in the alpha and low-beta bandwidths (alpha: from 0.143 ± 0.129 to 0.132 ± 0.129, *p* = 0.035; low-beta: from 0.117 ± 0.039 to 0.086 ± 0.03, *p* = 0.0001), whereas no significant changes were found in the other ones (*p* > 0.05). The discharge rate (DR) and the Force Covariance (CovF%) were not significantly affected by acute WBV exposure (*p* > 0.05).

**Discussion:** According to the significant effects found in alpha and low-beta bandwidths, which reflect sensorimotor integration parameters, accompanied by no differences in the DR and CovF%, the present results underlined that possible neural mechanisms at the base of the previously reported performance enhancements following acute WBV are likely based on sensorimotor integration rather than direct neural drive modulation.

## 1 Introduction

Over the past years, the principal approaches to enhance gross motor control and specific adaptations in different performances have been widely studied in healthy and non-healthy populations (Cardinale and Bosco, 2003; Tamini et al., 2020; Minhaj T, 2022). Acute exposure to whole-body vibration (WBV) has been shown to enhance muscle strength and power (Delecluse, Roelants, and Verschueren, 2003; Rees, Murphy, and Wastford, 2009; Coelho-Oliveira, 2021), posture (Orr, 2015), flexibility (Guedes-Aguiar et al., 2023)and coordination (Cochrane et al., 2010; Brent Feland et al., 2021). These improvements have been associated with enhanced neural excitation, likely linked to central nervous system modulation at spinal and supraspinal levels (Cochrane et al., 2010). In addition, other neural mechanisms may be responsible for the previously mentioned improvements, such as altered motoneuron excitability, synergist, and antagonist coactivation, spindle sensitivity, motor unit (MU) recruitment thresholds, and synchronization (Cardinale and Bosco, 2003). It has been suggested that muscle spindles are massively targeted by mechanical vibration in both animals and humans, which may be crucial for explaining part of the effects mentioned above, delivering a solid excitatory input to targeted muscle motoneurons and their synergists (Proske and Gregory John, 1999; Cardinale and Bosco, 2003; Krutki et al., 2022). However, specific neural mechanisms are not utterly clear. Short-term changes in the interaction between the sensorimotor cortex and spinal neurons have been described using corticomuscular coherence in different conditions (Semmler et al., 2004; Tecchio et al., 2006; Ushiyama et al., 2011; Hug et al., 2021). According to previous findings (Semmler et al., 2004; Farina and Negro, 2015), coherence can be described as the synchronization between motor units, measured from pairs of motor unit discharge trains through a correlation analysis in the frequency domain (Conway et al., 1995; Ushiyama et al., 2011). Moreover, the same correlation analysis in the domain of time (Proportion of Common Synaptic Input—PCSI) refers to the common synaptic input to all motor neurons as a percentage ratio to the independent one, as described in schematic models (Luca and Erim, 1994; Farina and Negro, 2015). Thus, a virtual correlation of 0.6 refers to 60% of PCSI.

Coherence analysis is based on a frequency spectrum organized into the following bandwidths: delta (0–5 Hz), alpha (5–15 Hz), beta (15–35 Hz), and piper (35–50 Hz) (Zandvoort et al., 2019; Coffey et al., 2021; Wijk et al., 2022; Vecchio et al., 2023). The present study focuses on alpha and beta band analysis due to their high association with afferent pathways and corticospinal parameters (Conway et al., 1995). Furthermore, an additional distinction can be made within the beta bandwidth, discerning between low-beta (≈15–21 Hz) and high-beta (≈21–35 Hz) ranges (Oswal et al., 2016; Wijk et al., 2017). Technological advances, such as the High-Density surface Electromyography technique (HDsEMG) (Holobar et al., 2009), have allowed researchers to develop more accurate and reliable tools for conducting motor unit studies and coherence analysis (Negro, Yavuz, and Farina, 2016). In the present study, the within-muscle common neural drive was studied through coherence (Farmer et al., 1993; Hug et al., 2021) applied to motor unit spike trains identified from an isometric dorsiflexion task in sustained low-intensity contractions in the same muscle. In accordance with recent shreds of evidence (Lecce et al., 2023), acute WBV elicits no significant effects on the discharge rate and recruitment threshold at different %MVF. Thus, sensorimotor integration may be the basis for the abovementioned results, which were studied by associating several neural parameters with performance outcomes (Cardinale and Bosco, 2003; Cochrane et al., 2010). Based on previous hypotheses concerning acute WBV exposure (Delecluse, Roelants, and Verschueren, 2003; Orr, 2015; Brent Feland et al., 2021), cortico- and corticospinal-based changes in neural control following acute vibratory stimuli were expected. Coherence analysis may be a reliable tool to investigate these parameters, giving a deeper insight into the acute effects of WBV on neural control. The present study aims to determine the primary and potential sources of neural modulation following acute WBV exposure through coherence analysis to clarify the potential mechanisms at the base of the effects reported in the studies mentioned above.

## 2 Methods

### 2.1 Participants and ethical approval

Fourteen recreationally active men (mean ± SD; age, 25 ± 2.1 years; BMI = 23.9 ± 1.3 kg m^2^; Maximal voluntary force (MVF): 324.36 ± 41.26 N) took part in the study. Participants were given an alphanumerical code. One volunteer withdrew from the study. The exclusion criteria included: 1) history or signs of neurological or neuromuscular diseases; 2) having undertaken limb surgery 3) being currently under any pharmacological treatment; 4) smoking habit. Participants signed a written informed consent outlining experimental procedures and potential side effects before participating in the study. The experiments were conducted following the Declaration of Helsinki. The protocol was approved by the University of Milan Ethics Committee “Politecnico” (process number 09/2021).

### 2.2 Experimental design

Volunteers visited the laboratory on two occasions. During the first visit, participants did a familiarization with the protocol, performing maximal isometric voluntary contractions (MVCs) and steady isometric contractions at the resulting 10%MVF. In the second visit, after a standardized warm-up (3 × 30 low-intensity isometric contractions separated by 30 s), volunteers performed three MVCs separated by 3 min to minimize fatigue. Participants received strong verbal encouragement to achieve their maximum during each contraction. The MVF was set as the peak force value across the 3 MVCs. After 5 min of rest, participants performed a 1 min steady contraction at the target value (10%MVF). They were asked to exert force accurately matching the continuous path on a monitor 1 m from their eyes. Thirty seconds after the first sustained contraction, WBV was applied (see 2.3 WBV protocol); a second 10%MVF contraction was performed right after the end of the vibrating protocol. The myoelectrical signals were recorded exclusively within contractions through the high-density surface EMG (HDsEMG) from the tibialis anterior (TA) of the dominant leg, which was identified by asking participants which leg they use to kick a ball (Nuccio et al., 2021b). TA was chosen due to the mechanical vibrating application proximity (Lecce et al., 2023). Participants were asked to refrain from strenuous exercise and caffeine consumption in the 48 h before the testing sessions.

### 2.3 WBV protocol

**FIGURE 1:**
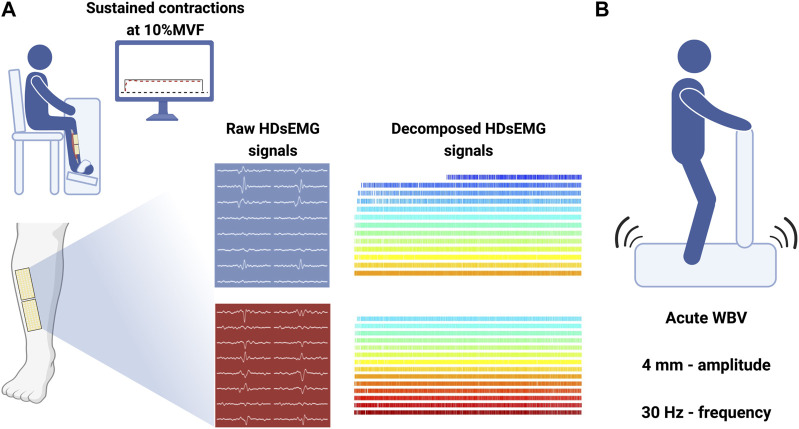
Experimental setup. **(A)** Custom-made ergometer for TA force and HDsEMG signal recordings with sustained path displayed on a monitor placed 1 m from participants’ eyes. Grids are placed above and below the IZ over the TA belly, and decomposed signals were obtained from BSS applied to raw data. **(B)** Vibratory stimuli (4 mm; 30 Hz) were delivered through a platform; participants were placed upright. Made with Biorender.com.

Volunteers stood upright and barefoot on a vibrating platform (Globus, Physioplate Fit, 230V/50Hz, Codognè [TV]) (Figure 1). The mechanical vibration frequency was set to 30 Hz and the amplitude to 4 mm (Cormie et al., 2006). Participants were exposed to a 1 min WBV bout. They were asked to maintain a static body position in a forefoot stance with a 10° knee angle (taking 0° as the anatomical position) and a 120° plantarflexion angle. Volunteers were instructed to place their hands on the platform handle, directing their head and eyes forward and distributing their weight equally on both feet. Once participants were correctly positioned, the vibrating stimulus started.

### 2.4 Force signal recording

The experimental setup comprised a vertically positioned custom-made ankle ergometer (OT-Bioelettronica, Turin, Italy). Volunteers were seated on a chair with the dominant leg in the ankle ergometer with straps at the foot, ankle, and knee. The hip and the knee were flexed at ∼90° and the ankle at ∼100° (plantarflexion). The foot and the ankle were maintained with straps on an adjustable footplate connected in series with a calibrated load cell (CCt transducer s.a.s. Turin, Italy). The force signal, recorded from the load cell, was amplified (x200; 1 channel force amplifier Forza-B, OT-Bioelettronica, Turin, Italy) and synchronized with the electromyogram and sampled at 2048 Hz with an external analog-to-digital (A/D) converter (EMG-400, OT-Bioelettronica, Turin, Italy). Since the sustained contraction at 10%MVF needed to be visually guided, a steady pattern was shown to participants during the contraction, with a minimum/maximum error of 3% MVF.

### 2.5 High-density surface electromyography recordings (HDsEMG)

HDsEMG signals were recorded from the TA with a pair of two-dimensional adhesive grids of 64 electrodes [13 rows x 5 columns; gold-coated; diameter: 1 mm; inter-electrode distance (IED): 8 mm; OT-Bioelettronica, Turin, Italy]. After skin preparation (shaving, light skin abrasion, and 70% ethanol cleansing), the muscle perimeter was identified through palpation and marked by a surgical pen. The grids orientation was based on recordings from a 16-electrodes array (IED 5 mm, OT-Bioelettronica, Turin, Italy), identifying the TA innervation zone (IZ) and estimating the direction of the fibers, as described in (Vecchio et al., 2019). The IZ was located by identifying the point of inversion in the propagation direction of action potentials proximally (toward TA proximal tendon) and distally (toward TA distal tendon) along the electrode column. Once the IZ was found, one grid was placed over the TA distal portion, and the other was proximally placed (Figure 1), both parallelly to the lateral tibial margin using a disposable biadhesive with layer holes adapted to the HDsEMG grids (SpesMedica, Battipaglia, Italy). The foam layer holes were filled with a conductive paste (SpesMedica, Battipaglia, Italy) to ensure skin-electrode contact. Reference electrodes were positioned on the styloid process of the ulna, on the tibial tuberosity, and on the medial malleolus (Lecce et al., 2023). HDsEMG signals were recorded in monopolar mode and converted to digital data by a 16-bit multichannel amplifier (EMG-Quattrocento, OT Bioelettronica, Turin, Italy). The HDsEMG signals were amplified (x150), sampled at 2048 Hz, and band-pass filtered (10–500 Hz) before being stored for offline analysis.

### 2.6 Force and HDsEMG analysis

**FIGURE 2:**
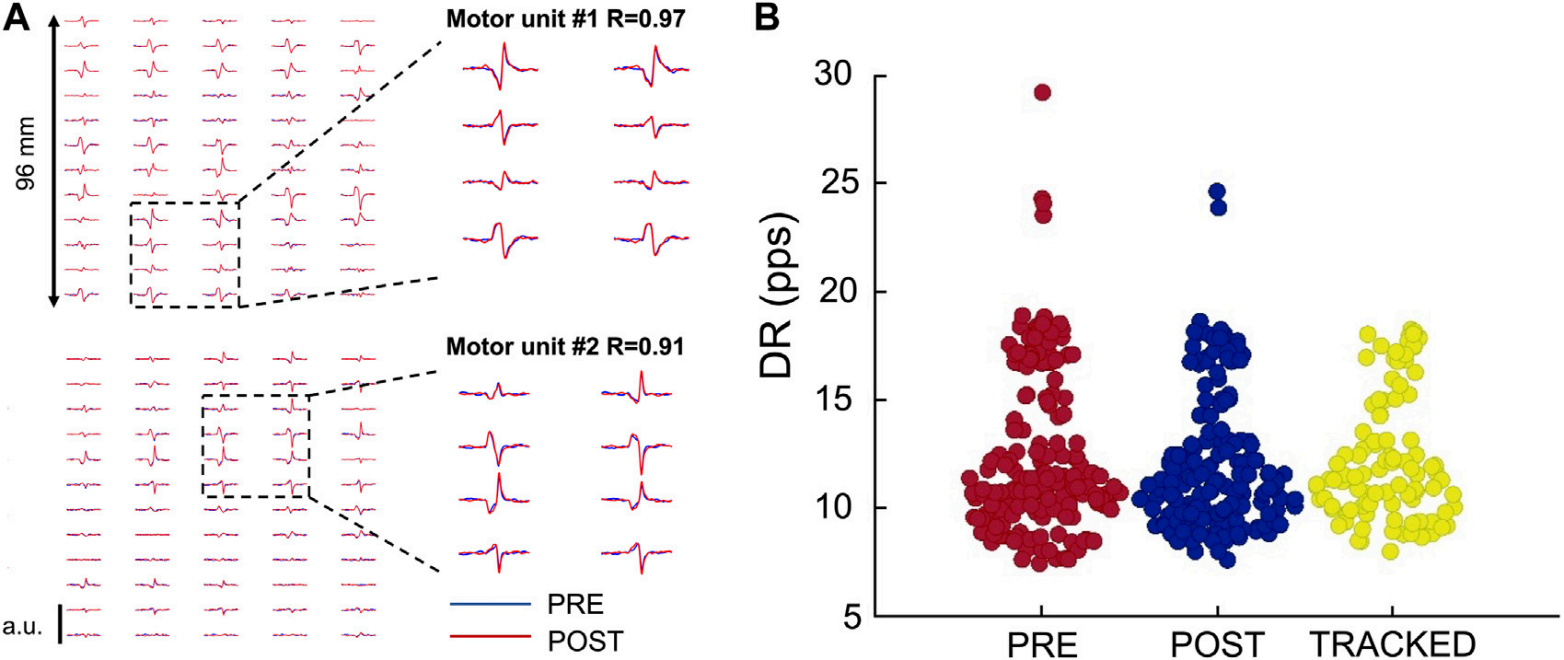
Motor unit tracking across conditions. **(A)** Action potentials of two tracked motor units recorded through HDsEMG. **(B)** Swarm plot of all the identified motor units Pre- and Post-WBV plus tracked MUs as a function of the discharge rate (pps) on the ordinate axis. Identified motor units are 358 (PRE: 181; POST: 177); Tracked motor units are 98.

The HDsEMG signals were decomposed into Motor Unit Spike Trains (MUSTs) by using the Convolution Kernel Compensation (CKC) decomposition framework, an algorithm relying on the blind source separation (BSS) method (Germer et al., 2021; A Holobar and Zazula, 2007), implemented in the DEMUSE tool software working on MATLAB (MathWorks Inc. Natick, United States). This decomposition procedure can identify motor unit discharge times over a broad range of forces (Figure 2). An experienced investigator manually analyzed the identified MUs, retaining only those characterized by a high pulse-to-noise ratio (A Holobar et al., 2014). MUs with a pulse-to-noise ratio <30 dB or a discharge time separated by more than 2 s were excluded from the analysis. Motor units were tracked across the pre-and post-WBV. The reliability of the motor unit tracking is based on the correlation value of the two-dimensional action potential waveforms (Vecchio et al., 2019) and has been widely validated (A Holobar and Farina, 2014). Therefore, *r* < 0.8 was retained as a good correlation value, and all motor units showing a lower r-value were discarded. Force steadiness was quantified by using the coefficient of variation of force (CovF%), defined as the percent ratio between the standard deviation and the mean force (SD/mean x 100) calculated over the 30 s period starting from the 15th second, considered in 1 s windows as described in previous studies (Bazzucchi et al., 2004; Castronovo A. Margherita et al., 2015; Inglis and Gabriel, 2021). The discharge rate (DR), which is the frequency of discharge times identified by the decomposition, and the interspike interval (ISI), which is the time between two consecutive spikes, were identified during the offline analysis, as described in previous studies (Nuccio et al., 2021a).

### 2.7 Within-muscle coherence

**FIGURE 3:**
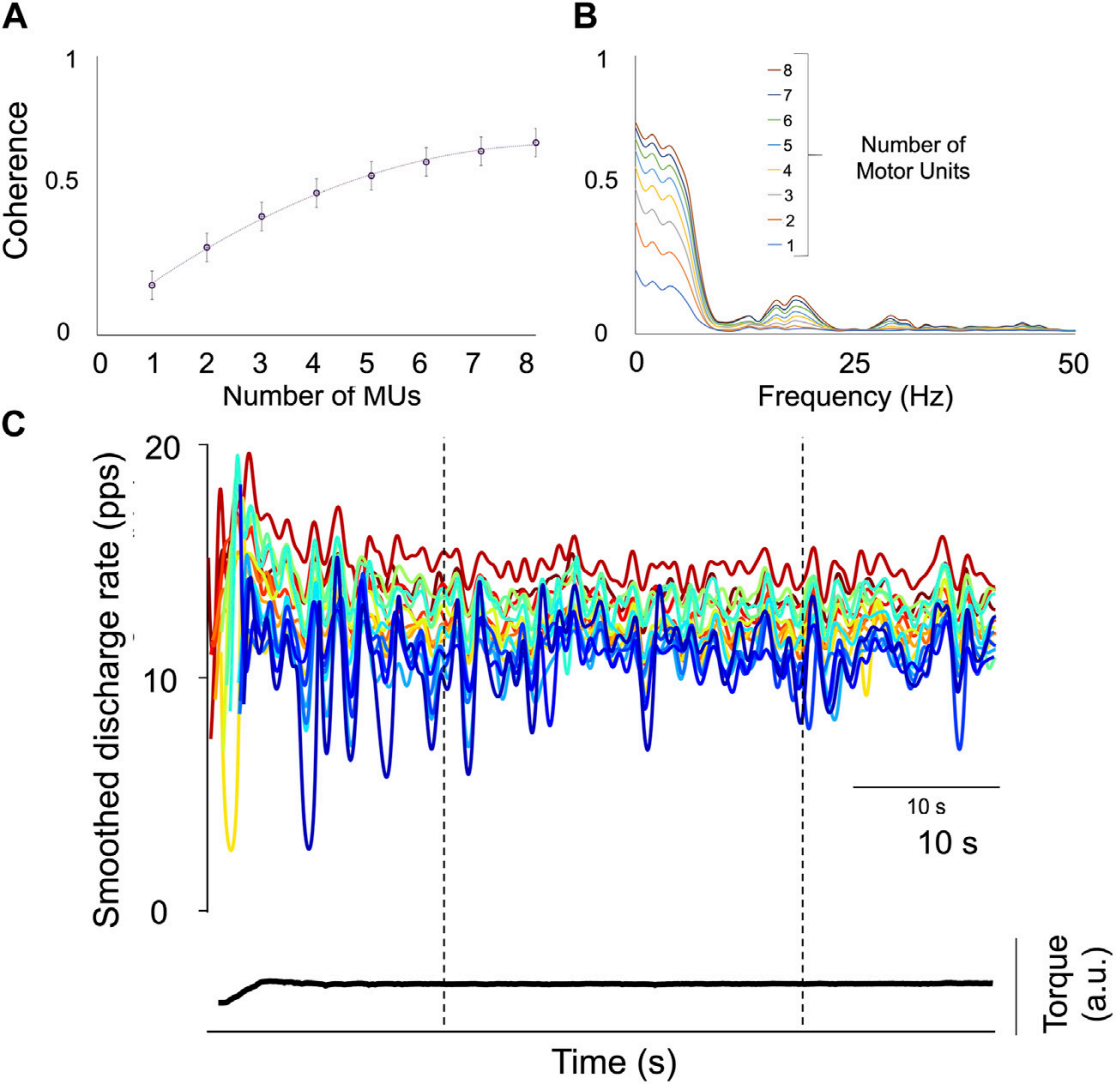
Assessment of the Proportion of Common Synaptic Input (PCSI). **(A)** The relationship between the mean coherence value (±SD) in the delta bandwidth (0–5 Hz) and the increasing number of motor units are displayed. The rate of change is considered an index of the PCSI. **(B)** Individual results of coherence analysis performed on two cumulative spike-trains (CSTs) with an increasing motor unit number (1–8). **(C)** Displays the smoothed discharge rate of 17 motor units from the tibialis anterior (TA) of Participant 1 during a 10%MVF sustained contraction; the space defined by the two broken lines represents the window time in which coherence was analyzed. The total contraction time is 60 s.

Within-muscle correlation (within-muscle coherence) has been widely used to assess neural connectivity between motor units from the same muscle (Castronovo A. Margherita et al., 2015; Hug et al., 2021). The degree of correlation between spike trains was calculated using coherence analysis on the unfiltered composite spike trains (summation of individual spike trains) in the frequency domain (Farina, Negro, and Jiang, 2013). As described above, the motor unit number used for coherence calculation correlates with the final coherence value, which monotonically increases (Castronovo AnnaMargherita et al., 2015; Dideriksen, 2018; Hug et al., 2021). Therefore, the function representing the coherence value as a function of the motor neuron spike trains is a monotonically increasing function (Figure 3). In particular, the more the common input, the greater the rate of coherence increase. Thus, for the same motor unit number involved in the estimation, differences in coherence represent differences in the strength of common input. In the present study, only those contractions with a minimum number of 6 motor units were analyzed. In addition, the coherence values were calculated across conditions in tracked motor units; as a result, the same motor unit number was involved in the analysis between the Pre- and the Post-WBV. The coherence profiles were calculated across the following frequency bands 1–5, 5–15, 15–21, 21–35, and 35–50 Hz. Coherence calculated at a given frequency represents the correlation between two signals, ranging between 0 (no correlation) and 1 (perfect correlation).

In the present study, those motor units which were decomposed at the beginning and the end of the sustained tasks were excluded due to the recruitment phase, which was non-visually guided. In addition, coherence was analyzed only in the central 30 s window within the 1 min of contraction, excluding the initial recruitment portion (Figure 3). The proportion of common synaptic input (PCSI) to motor neurons was estimated using a method that was validated in numerical simulations based on a model of motor neuron populations receiving common and independent inputs (Negro et al., 2016). The PCSI was estimated in delta (0–5 Hz) bandwidth.

### 2.8 Statistical analysis

A statistical power analysis was performed *a priori* to determine the sample size (G*power software version 3.1.9.4; *α* = 0.05, power = 0.80, effect size = 0.4; the total sample size needed was 12). The data normality distribution was assessed using the Kolmogorov- Smirnov test. When the variables were not normally distributed, nonparametric statistical tests were used, observing specific guidelines for the nonparametric approach (Maris and Oostenveld, 2007). When needed, the assumption of sphericity was verified by Mauchly’s test, which was never violated. Since coherence values across bandwidths showed a non-normal distribution, related-samples Wilcoxon Signed Rank Tests were performed. One-way ANOVA was used to evaluate changes in the proportion of common synaptic input (PCSI), ISI, and the covariance of force (CovF%). The statistical calculations were performed using SPSS 25.0 (IBM Corp., Armonk, NY, United States). A *p* < 0.05 was considered a statistically significant result. Data are reported as the Mean ± SD in the text.

## 3 Results

### 3.1 Motor unit decomposition and tracking

An average of 12.9 ± 4.9 motor units were identified from each participant, with a total of 362 motor units. Of these, 98 motor units were tracked (roughly 27%), with an average number of 7.3 ± 2.6 tracked motor units across the 13 participants (Figure 2). The average two-dimensional correlation for the tracked action-potential waveforms across the two contractions (PRE- and POST-WBV) was 0.95 ± 0.02. This analysis is based on the correlation values of the two-dimensional action-potential waveforms (Martinez-Valdes et al., 2017).

### 3.2 Within-muscle coherence

**FIGURE 4:**
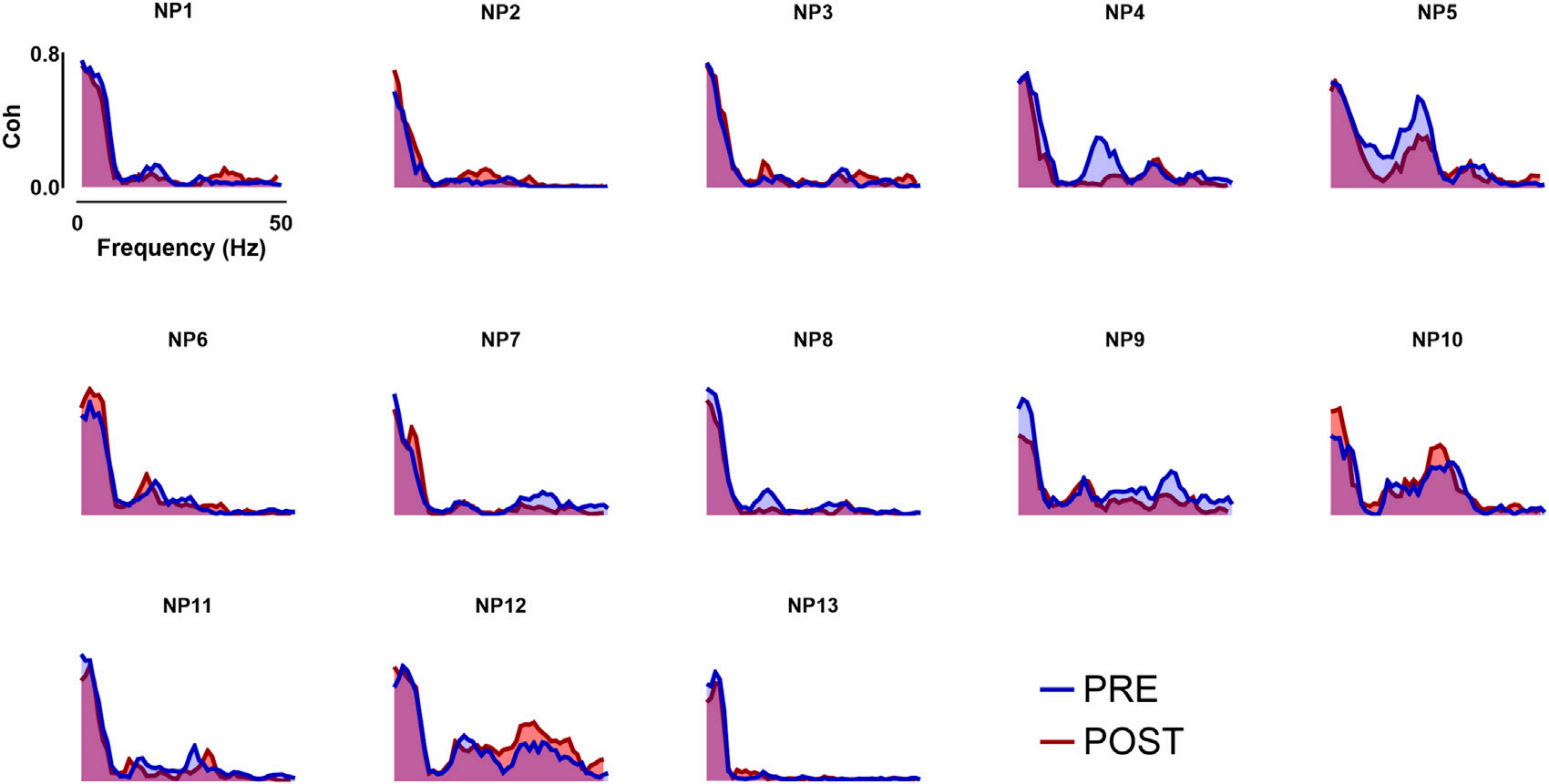
Coherence analysis across all frequency bands taken into analysis. In the image above, the coherence profiles of all participants for both Pre- and Post- WBV exposure are displayed. Coherence analysis was performed in the frequency domain within the whole frequency band (0–50 Hz).

**FIGURE 5:**
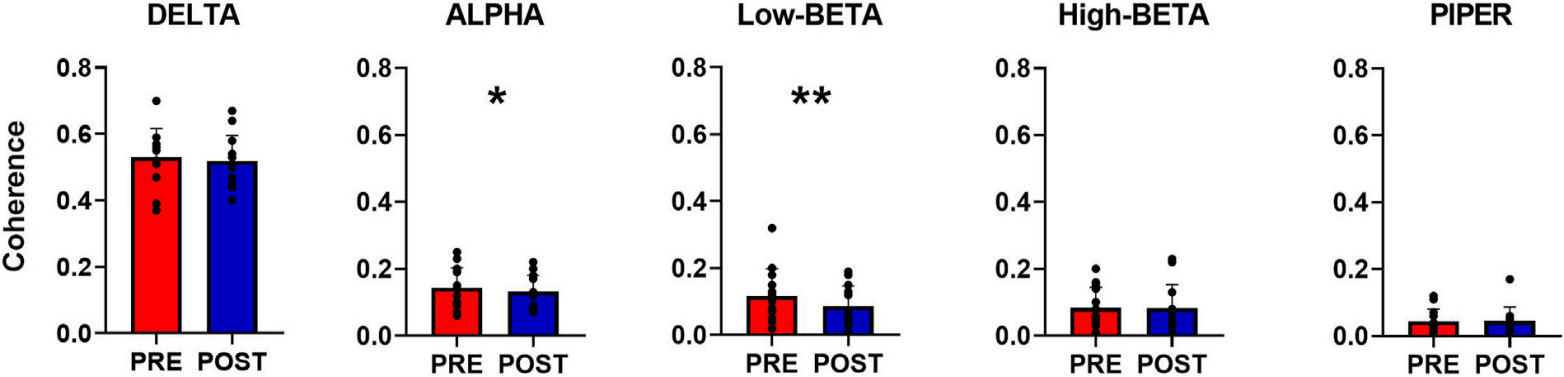
Overall coherence analysis. The bar plot displays the differences between pre-and post-WBV exposure in all the identified frequency bands. Data are reported as the mean ± SD, *p < 0.05, **p < 0.01.

The within-muscle coherence analysis was performed on tracked motor units. The PCSI was calculated from the relationship between the average coherence values in the delta band (0–5 Hz) and the number of motor units involved in the estimation (see methods). No significant effects were found for the PCSI (from 0.50 ± 0.15 to 0.49 ± 0.16, *p* = 0.747). However, statistically significant differences were observed in the mean coherence for alpha and low-beta bandwidths (alpha: from 0.143 ± 0.129 to 0.132 ± 0.129, *p* = 0.035; Low-Beta: from 0.117 ± 0.039 to 0.086 ± 0.03, *p* = 0.0001), whereas no significant effects were found in the other bandwidths (delta: from 0.532 ± 0.08 to 0.519 ± 0.08, *p* = 0.247; High-Beta: from 0.086 ± 0.044 to 0.0083 ± 0.038; *p* = 0.369; Piper: from 0.045 ± 0.02 to 0.046 ± 0.021, *p* = 0.778). Coherence results are reported in Figure 4 and Figure 5.

### 3.3 Neuromechanical changes after acute WBV

**FIGURE 6:**
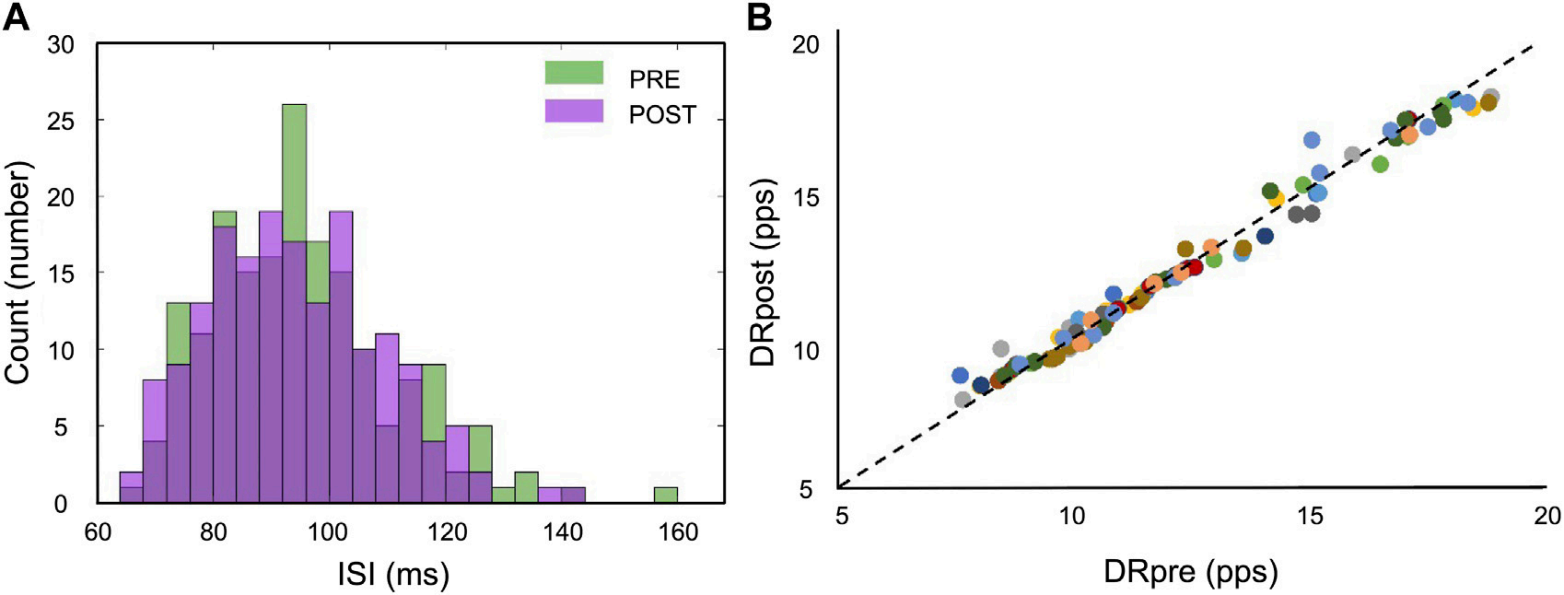
Motor unit discharge properties. **(A)** Interspike intervals (ISIs) distribution is displayed through a histogram plot. No significant differences were found between the Pre- and Post-WBV. **(B)** Scatter plot of the discharge rate (DR) for the same motor unit for all participants, marked with a different color each.

After acute WBV exposure, discharge rate (DR) failed to show a significant change (from 12.22 ± 3.05 to 12.28 ± 2.93 pps, *p* = 0.181). In addition, the ISI effects resulted non-statistically significant (from 95.39 ± 15.7 to 94.08 ± 14.8 m, *p* = 0.421). Discharge properties results are reported in Figure 6. In addition, force steadiness measured as the covariance of force (CovF%) showed a non-significant decrease compared to the PRE (from 2.00 ± 0.76 to 1.98 ± 0.87, *p* = 0.952).

## 4 Discussion

The present study analyzed the common synaptic input (CSI) to the tibialis anterior motor unit pool in sustained contractions at 10% MVF before and after acute whole-body vibration exposure. Compared to other studies (Martinez-Valdes et al., 2017), where HDsEMG and motor unit tracking were used, we tracked a similar percentage of motor units after the offline decomposition. The overall coherence analysis was performed across different frequency bands. It was found moderately common drive to the motor neuron pool (see results, 0–5 Hz bandwidth). This finding aligns with previous studies (Castronovo A. Margherita et al., 2015; Hug et al., 2021), suggesting that motor neurons innervating single muscles receive most of their input from a common source. In addition, the common drive seemed not to be affected by acute WBV. Even though coherence was not influenced in the low- and high-frequency range, the sensorial and corticospinal bandwidth (0–15 Hz; 15–21 Hz) showed significant changes after the acute vibrating stimulus, reflecting a modification in the sensorimotor integration bandwidth. These findings suggest that changes in performance described in previous studies may be based on a different sensorimotor integration rather than direct neural drive modulation. On the other hand, force control analysis showed non-significant effects after acute WBV, which can be explained by the assumption that force control parameters are not utterly dependent on sensorimotor integration, especially in low-intensity contractions. Indeed, changes in the low-frequency band (Delta, 0–5 Hz), which represents the neural drive, are directly associated with force control (Castronovo AnnaMargherita et al., 2015). Thus, acute whole-body vibration exposure may influence other mechanical parameters, such as maximum force and power output (Cardinale and Bosco, 2003). Nevertheless, according to previous studies (Baldissera, Cavallari, and Cerri, 1998; Castronovo AnnaMargherita et al., 2015), a change in force output is associated with an approximately corresponding modification in the neural drive to the muscle, filtered by the average twitch of the active motor units. In the present study, the non-significant effects in the neural drive are accompanied by an almost unalerted ISI and a non-significant decrease in the mean discharge rate (DR), reflecting non-significant effects in both the common and independent inputs. These results suggested that acute whole-body vibration exposure did not elicit changes in motor unit discharge properties in low-intensity sustained contractions.

Moreover, alpha and low-beta bandwidth changes reflect the interaction between cortical and spinal pathways (Witte et al., 2007), especially in low-force sustained contractions used in the present study protocol. Previous studies have described similar effects, showing that increased coherence in beta-bandwidth is associated with fatiguing task responses (Kattla and Lowery, 2010; Ushiyama et al., 2011). An increased motor unit synchronization may be considered a neural mechanism to optimize mechanical parameters for task-dependent demands, such as force control in fatiguing conditions or perturbated tasks, such as mechanical oscillations. Our results revealed a significant decrease in coherence values in the alpha and low-beta bandwidth, indicating a reduction in the common synaptic input in favor of the independent one. Therefore, it can be hypothesized that a physiological vibration-induced adjustment may be responsible for facilitating motor unit independent input. Possible between-muscle coherence variations may occur after WBV exposure, more likely in synergistic muscles during compound-movement tasks. Another plausible hypothesis aligned with previous results is represented by the potential interaction between vibratory stimuli and muscle primary endings (Ia), which may result in an altered muscle spindle activity (Proske and Gregory John, 1999; Krutki et al., 2022), reflected in reduced alpha bandwidth coherence.

The present study reported a coherence decrease in the low-beta bandwidth between cumulative trains of motor unit discharges with acute WBV exposure. Acute whole-body vibration exposure did not seem responsible for changes in discharge properties and force control in low-intensity sustained contraction. In contrast, acute WBV elicited changes in the sensorial and corticospinal bandwidths (alpha, 5–15 Hz; low-beta, 15–21 Hz), which may be responsible for changes in sensorimotor integration, confirming previous hypotheses (Cardinale and Bosco, 2003; Delecluse, Roelants, and Verschueren, 2003; Orr, 2015; Brent Feland et al., 2021). Results were interpreted as reduced coordination between motor units reflected by the significant decrease in the alpha and low-beta bandwidths.

The PCSI results are based on esteemes obtained in post-acquisition analysis, which represents the main limitation of the present study. Moreover, isometric contractions are mandatory for HDsEMG acquisition and data analysis; thus, motor unit behavior and coherence in dynamic conditions remain unexplored. On the other hand, the present technique confers a broader perspective of electromyographical analysis than classical EMG due to the opportunity to identify and track motor units, providing a deeper insight into neuromuscular behavior in several conditions, such as those explored in the present paper.

In conclusion, the present results described the neuromuscular response to acute WBV (30 Hz; 4 mm; 1 min), showing that principal effects are based on the modulation of sensorial and sensorimotor pathways. Therefore, the effects reported in previous studies may be based on a sensorimotor integration variation rather than direct neural drive effects. Furthermore, according to the results of both coherence and discharge properties analyses, WBV did not elicit effects on the neural drive and force steadiness.

## Data Availability

The raw data supporting the conclusion of this article will be made available by the authors, without undue reservation.
